# Symptoms and patterns of symptom propagation in incipient ischemic stroke and migraine aura

**DOI:** 10.3389/fnhum.2022.1077737

**Published:** 2023-01-13

**Authors:** Adrian Scutelnic, Jacqueline Bracher, Lukas A. Kreis, Morin Beyeler, Urs Fischer, Marcel Arnold, Heinrich P. Mattle, Simon Jung, Christoph J. Schankin

**Affiliations:** ^1^Department of Neurology, Inselspital, Bern University Hospital, University of Bern, Bern, Switzerland; ^2^Department of Neurology and Stroke Centre, University Hospital Basel and University of Basel, Basel, Switzerland

**Keywords:** migraine aura, ischemic stroke, transient ischemic attack, dynamics, acute neurological deficit

## Abstract

**Background and objectives:**

Taking a detailed history of symptoms is important for differentiating incipient ischemic stroke and migraine aura. The aim of our study is to describe in detail symptom type and temporal pattern of symptom evolution (i.e., symptom succession and the time lapse between symptoms) and to identify differentiating clinical features in patients with ischemic stroke and migraine with aura.

**Methods:**

Consecutive patients with ischemic stroke and migraine with aura were interviewed using a structured questionnaire. Stroke diagnosis was confirmed by imaging and migraine with aura was diagnosed according to the current criteria of the International Headache Society. Wake-up strokes and patients with severe cognitive deficits were excluded.

**Results:**

In stroke patients and migraine patients, respectively, 50/78 (64%) vs. 123/326 (37%) had one, 18 (23%) vs. 127 (38%) had two, 5 (6%) vs. 69 (21%) had three, 2 (2%) vs. 4 (1%) had four, and 3 (3%) vs. 3 (1%) had five visual symptoms. In respect of sensory symptoms, 76/145 (52.4%) vs. 116/175 (66%) reported paresthesia and 92/145 (63.4%) vs. 132 (75%) numbness. Looking at the beginning, visual symptoms were the first symptom more often in migraine aura than in ischemic stroke (72.1 vs 18.8%, *P* < 0.001; PPV 86.8%). Sensory (29 vs 13.9%, *P* = 0.001; PPV 54.8%) and motor symptoms (20.5 vs 1.4%, *P* < 0.001; PPV 88.9%) were the first symptom more frequently in ischemic stroke. Of patients with consecutive symptoms, 39 of 201 (19%) compared to 34 of 117 (29%) (*P* = 0.02; PPV 46.6%) reported at least two simultaneous symptoms. A time lapse between symptoms of < 1 min (18.6 vs 6.3%, *P* < 0.001; PPV 57.1%) and > 360 min (15.8 vs 0%, χ^2^ = 39.61, *P* < 0.001; PPV 100%) was more frequent in stroke whereas a time lapse between 5 and 60 min was more frequent in migraine aura (41.1 vs 68.7%, χ^2^ = 23.52, *P* < 0.001; PPV 78.7%).

**Conclusion:**

There is a significant overlap in the clinical presentation of stroke and migraine aura. In particular, a substantial proportion of patients in one group had symptoms that are traditionally attributed to the other group. This study highlights the similarities and differences between symptoms of ischemic stroke and migraine aura and challenges our reasoning in daily clinical practice.

## Introduction

In clinical practice, differentiating migraine aura from incipient ischemic stroke might be challenging, in particular due to the heterogeneous and often overlapping clinical manifestation: (Viana et al., 2016, 2017). Migraine with aura (MwA), which typically contains positive, slowly occurring, spreading and consecutive symptoms of 5–60 min duration, may contain negative symptoms, symptoms with sudden onset and symptoms occurring simultaneously; symptoms in migraine aura may last longer than 60 min according to the current diagnostic criteria; TIA and ischemic stroke may manifest with migraine aura-like symptoms, i.e., positive irritative symptoms, symptoms with gradual onset and symptoms occurring in succession (Fisher, 1968; Olesen et al., 1993; Cohen et al., 2002).

The type of symptoms and temporal aspects of migraine aura have been described previously (Bücking and Baumgartner, 1974; Kelman, 2004; Viana et al., 2016, 2017). However, the description of ischemic stroke symptoms in regard to type, spreading and succession of symptoms is limited to small case series (Fisher, 1968; Cohen et al., 2002). A detailed characterization of symptoms at stroke onset might help to better understand the spectrum of ischemic brain events and aid in the clinical diagnosis and management.

Previously, we have demonstrated a surprisingly high overlap of symptoms between the ischemic stroke and migraine aura (Scutelnic et al., 2022). Here, we present a more detailed description of the two groups regarding symptom type, pattern of symptom spreading and succession, and the temporal lapse between symptoms. In addition, given the different pathophysiology, we aimed to identify clinical features that might differentiate ischemic stroke from migraine aura.

## Materials and methods

These have been previously reported (Scutelnic et al., 2022). In short, in this single center study consecutive patients with acute ischemic stroke confirmed by imaging [2013 AHA definition (Sacco et al., 2013)] and patients with definite migraine with aura (MwA) diagnosed according to International Classification of Headache Disorders 3rd edition (ICHD-3) (Headache Classification Committee of the International Headache Society [IHS], 2018) treated at our stroke center and outpatient headache clinic between 03/2019 and 08/2021 have been interviewed using a structured questionnaire. There were multiple answer options for every question (e.g., type of visual disturbance), and the participant had to choose the most suitable option to describe the own symptoms. If the participant found that no option was suitable or sufficient, we recorded the participant’s description in his/her own words.

The statistical analysis was performed in STATA/MP 16.0, StataCorp LLC. Categorical data are presented in counts. The medians are presented as 25–75% interquartile ranges (IQR). For categorical variables χ^2^ or one-sided Fisher exact test have been used, as appropriate. A *P* of < 0.05 was considered as statistically significant. The time lapse between pairs of symptoms were analyzed. For example, if a patient had three consecutive symptoms, the time lapses between first and second (first pair) and second and third symptoms (second pair) were assessed. If multiple symptoms occurred simultaneously after a preceding symptom was reported, they were judged as a single pair of symptoms. For selected variables which significantly differed between the groups, positive and negative predictive values (PPV, NPV) were calculated.

## Results

Four hundred thirty one patients with ischemic stroke were interviewed, of which 81 had to be excluded due to wake-up symptoms leaving 350 patients for analysis. Three hundred forty three patients with definite migraine with aura (MwA) fulfilling the ICHD-3 C criteria were included. The median age was 71 (IQR 62–78) in the stroke and 29 (IQR 28–52) in the MwA group. There were 61% men in the stroke group and 69% women in the MwA group. Median NIHSS was 1 (IQR 0–3, range 0–13) in the stroke group. The interview took place a median of 2 (IQR 1–3) days after the stroke and of 28 days (IQR 7–118) after the last aura. Fourteen patients with stroke (4% of 350) had a history of co-morbid MwA.

### Visual symptoms

**FIGURE 1:**
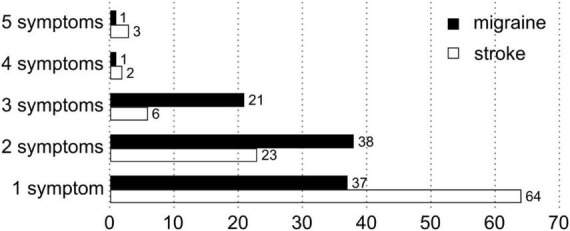
Distribution of number of visual symptoms (in %).

Seventy eight of 350 (22.3%) stroke patients and 326 of 343 (95%) MwA (χ^2^ = 377.2, *P* < 0.001) patients reported visual disturbance (Table 1 and Figure 1). Compared to patients with stroke (17/78, i.e., 20%), more patients with MwA (209/326, i.e., 64%, χ^2^ = 45.72, *P* < 0.001) reported answers that were different to the predefined options (Supplementary Tables 1, 2).

**TABLE 1:** Visual symptoms in patients with ischemic stroke and migraine aura.

	Ischemic stroke n/N (%)	Migraine with aura n/N (%)	*P*-value (χ^2^)
Blurry vision	30/78 (38)	81/326 (23)	0.01
Bright vision	11/78 (14)	154//326 (45)	<0.001
Overly colored	7/78 (9)	44/326 (13)	0.28
Zig-Zag lines	8/78 (10)	66/326 (19)	0.04
Scintillating scotoma	22/78 (28)	168/326 (49)	<0.001
Dark vision/anopia	28/78 (35)	81/326 (23)	0.04

### Sensory symptoms

One hundred forty five of 350 (41.4%) stroke patients and 175 of 343 (51%) MwA patients reported sensory symptoms (χ^2^ = 0.46, *P* = 0.49). Paresthesia (76/145, i.e., 52.4% in stroke vs. 116/175, i.e., 66%, χ^2^ = 6.35, *P* = 0.01) and numbness (92/145, i.e., 63.4% vs. 132/175, i.e., 75%, χ^2^ = 5.4, *P* = 0.02) were common in both groups, but less common in stroke. Seven stroke patients gave an open answer additional to the predefined ones. Two reported an initial paresthesia followed by numbness, one had initial paresthesia followed by a cold feeling, two had a burning sensation, and two had a feeling of tension of the affected region. Nine MwA patients gave an open answer regarding the type of sensory symptoms: one had sensation of flowing water over the skin, one reported a strange feeling on the skin, one a burning sensation, one a painful sensation, one a pulling sensation, one a dull sensation, one had piloerection, one a furry sensation, and one could not describe the sensory disturbance.

### Speech symptoms

Speech symptoms were reported by 196 (56% of 350) stroke patients and 160 (46.6% of 343, χ^2^ = 6, *P* = 0.01) migraine patients. For a detailed description of speech symptoms of both groups, see Table 2 and Supplementary Tables 3, 4.

**TABLE 2:** Speech symptoms in patients with ischemic stroke and migraine aura.

	Ischemic stroke n/N (%)	Migraine with aura n/N (%)	*P*-value (χ^2^ or Fisher’s exact)
Difficulty finding the right words	63/196 (32)	119/160 (74)	<0.001
Difficulty understanding what others have said	7/196 (4)	18/160 (11)	0.005
Difficulty articulating words	150/196 (77)	73/160 (46)	<0.001
Unfamiliar words spoken	11/196 (6)	10/160 (6)	0.79
Neologisms	3/196 (2)	0	0.25

### Succession of symptoms

Two hundred and one of 350 (57.4%) stroke patients and 211 of 343 (61.5%, χ^2^ = 1.2, *P* = 0.27) MwA patients had more than one symptom. Of these, symptoms occurred less frequently in succession in stroke patients (117/201, 58.2%) than in MwA patients (201/211, 95.1%, χ^2^ = 80.2, *P* < 0.001). Visual symptoms (72.1 vs 18.8%, χ^2^ = 84.36, *P* < 0.001), were the first occurring symptoms more frequently in migraine aura than in ischemic stroke [PPV 86.8% (95%CI, 80.7–91.6), NPV 62.9% (54.7–70.6)]. In contrast, sensory [29 vs 13.9%, χ^2^ = 10.78, *P* = 0.001; PPV 54.8% (41.7–67.5), NPV 67.6% (61.5–73.3)] and motor symptoms [20.5 vs 1.4%, χ^2^ = 34.43, *P* < 0.001; PPV 88.9% (70.8–97.7), NPV 68.3% (62.6–73.6)] were more frequently the first occurring symptom in ischemic stroke. There was no significant difference of speech disturbance as first occurring symptom (11.9 vs 6.9%, χ^2^ = 2.3, *P* = 0.12). Of patients with consecutive symptoms, 39 of 201 (19.4%) migraine patients compared to 34 of 117 (29%, χ^2^ = 4.86, *P* = 0.02) stroke patients reported at least two simultaneous symptoms [PPV 46.6% (34.8–58.6), NPV 66.1% (59.8–72)]. For a detailed description of patterns of symptom succession see Table 3 and Supplementary Tables 5, 6.

**TABLE 3:** Patterns of symptoms occurring in succession reported by patients with ischemic stroke as well as patients with migraine aura.

	Stroke n/N (%)	Migraine n/N (%)	*P*-value (χ^2^ or Fisher exact)
**Patterns of spreading beginning with visual symptoms**			
Visual → right motor	5/117 (4)	1/201 (0.4)	0.01
Visual → speech	4/117 (3)	36/201 (18)	<0.01
Visual → sensory	2/117 (2)	35/201 (17)	<0.01
Visual → sensory + motor + speech	2/117 (2)	4/201 (2)	0.86
Visual → sensory → speech	2/117 (2)	30/201 (15)	<0.01
Visual → sensory + motor	1/117 (0.8)	2/201 (1)	0.69
Visual → sensory + motor → speech	1/117 (0.8)	7/201 (3)	0.15
Visual → sensory → motor	1/117 (0.8)	2/201 (1)	0.89
Visual → speech → sensory + motor	1/117 (0.8)	5/201 (2)	0.69
**Patterns of spreading beginning with sensory symptoms**			
Sensory → motor	9/117 (8)	3/201 (1)	<0.01
Sensory → speech	8/117 (7)	6/201 (3)	0.10
Sensory → visual	4/117 (3)	10/201 (5)	0.51
Sensory → motor → visual → speech	1/117 (0.8)	1/201 (0.4)	0.69
**Patterns of spreading beginning with speech disturbance**			
Speech → visual	3/117 (2)	6/201 (3)	0.82
Speech → sensory	2/117 (2)	5/201 (2)	0.64
**Patterns of spreading beginning with motor symptoms**			
Motor → visual	4/117 (3)	2/201 (0.9)	0.13
**Patterns of spreading beginning with multiple symptoms**			
Sensory + motor → speech	9/117 (8)	1/201 (0.4)	<0.01
Sensory + motor → visual → speech	2/117 (2)	2/201 (0.9)	0.46
Sensory + motor → visual	1/117 (0.8)	2/201 (0.9)	0.69

Of 117 stroke patients 93 (79.5%) and of 201 MwA patients 178 (88%) were able to report the time lapse between symptoms. In stroke 107 and in MwA 237 pairs of consecutive symptoms were identified (Table 4). In stroke a time lapse between symptoms of < 1 min [18.6 vs 6.3%, χ^2^ = 12.32, *P* < 0.001; PPV 57.1% (95%CI 39.4–73.7)], NPV 71.8% (66.5–79.8) and > 360 min [15.8 vs 0%, χ^2^ = 39.61, *P* < 0.001; PPV 100% (80.5–100), NPV 72.5% (67.3–77.3)] was more frequently reported. In MwA a time lapse between 5 and 60 min was more frequent [41.1 vs 68.7%, χ^2^ = 23.52, *P* < 0.001; PPV 78.7% (72.5–84.1), NPV 50% (37.4–54.7)]. In MwA the longest reported time lapse was 360 min and in ischemic stroke 7 days.

**TABLE 4:** Time lapse between symptoms in ischemic stroke and migraine aura.

	Stroke, n/N (%)	Migraine, n/N (%)	*P*-value (χ^2^ or Fisher exact)
≤1 min	20/107 (18.6)	15/237 (6.3)	<0.001
1.1–5 min	21/107 (19.6)	45/237 (18.9)	0.88
5.1–60 min	44/107 (41.1)	163/237 (68.7)	<0.001
60.1–120 min	1/107 (0.9)	11/237 (4.6)	0.08
120.1–180 min	2/107 (1.8)	1/237 (0.4)	0.18
180–360 min	2/107 (1.8)	2/237 (0.8)	0.36
>360	17/107 (15.8)	0/237	<0.001

### Similarities and differences of ischemic stroke and MwA symptoms

Ischemic stroke and MwA patients had patterns of wandering of sensory symptoms in common in 20/37 (54%) and 34/106 (32%) and of motor symptoms in 18/37 (48%) and 13/22 (59%) patients (Tables 5, 6). Patterns of sensory and motor symptoms and symptom-succession that were unique to one of the groups are shown in Supplementary Tables 7, 8. Spreading of sensory symptoms within one confined anatomical region (e.g., from one finger to another) was reported only by patients with MwA (10 of 175, 2.1%).

**TABLE 5:** Patterns of spreading of sensory disturbance reported by both patients with ischemic stroke and migraine aura.

	Stroke, n/N (%)	Migraine, n/N (%)	*P*-value (χ^2^ or Fisher exact)
**Spreading with beginning on the face, head and/or tongue**			
Face → arm + leg	1/37 (3)	2/106 (2)	0.59
Face → arm → leg	1/37 (3)	9/106 (8)	0.23
**Spreading with beginning in the arm**			
Arm → leg	9/37 (24)	2/106 (2)	<0.01
Arm → face	3/37 (8)	14/106 (13)	0.40
Arm → leg → face	2/37 (5)	2/106 (2)	0.27
**Spreading with beginning in the leg**			
Leg → arm → face	2/37 (5)	5/106 (5)	0.86
Leg → arm	2/37 (5)	1/106 (1)	0.16
Could not describe	0/37 (0)	11/106 (10)	0.04

**TABLE 6:** Patterns of spreading of motor symptoms reported by both patients with ischemic stroke and migraine aura.

	Stroke, n/N (%)	Migraine, n/N (%)	*P*-value (χ^2^ or Fisher exact)
**Spreading with beginning on the face and/or tongue**			
Face → arm → leg	1/37 (3)	5/22 (23)	0.01
**Spreading with beginning in the arm**			
Arm → leg	11/37 (30)	4/22 (18)	0.32
Arm → leg → face	3/37 (8)	2/22 (9)	0.62
**Spreading with beginning in the leg**			
Leg → arm → face	3/37 (8)	2/22 (9)	0.62
Could not describe	2/37 (5)	2/22 (9)	0.47

## Discussion

The main findings of our study are (1) the significant overlap of symptoms in incipient ischemic stroke and migraine aura, (2) the occurrence of visual symptoms as first symptom and the time lapse of 5–60 min between symptoms are predictive of migraine aura and (3) the occurrence of sensory and motor symptoms as first symptoms, the occurrence of two simultaneous symptoms, a time-lapse between symptoms of < 1 min and > 360 min are clinical features predictive for ischemic stroke.

We recently found the prevalence of positive irritative symptoms, such as scintillating scotoma or paresthesia, to be high in acute ischemic stroke (Scutelnic et al., 2022). Here, we further describe the type, spreading and succession of symptoms in acute ischemic stroke, but also identify clinical features that are characteristic for either group.

Although certain history elements are reported more frequently by migraine patients and are generally considered migraine-like, they may be reported by an important number of stroke patients as well. One example is the scintillating scotoma, which has been reported by 49% of MwA patients, but also by 28% of patients with stroke and visual disturbance. Regarding other elements of the history, such as sensory disturbance and time lapse between the symptoms, the similarities between stroke and MwA patients are striking: 52.4% stroke vs. 66% MwA patients reported paresthesia, 63.4% vs. 75% had numbness, and 19.6% and 18.9% of consecutive occurring symptoms in stroke and MwA had a time lapse of 5 min in between. Similarities also exist regarding the patterns of spreading of sensory and motor symptoms, but also of symptom succession, although the distribution of each pattern in MwA and ischemic stroke varies (Tables 3, 5, 6). Ischemia-induced cortical spreading depolarization (CSD), thrombus fragmentation and distal migration, gradual collateral failing and disinhibition phenomena might explain the overlap, as previously discussed (Scutelnic et al., 2022).

On the other hand, there are certain symptoms that have been more frequently described in ischemic stroke or migraine aura. The majority of patients with MwA (72.1%) described visual symptoms as first occurring, a clinical feature with high positive predictive value (86.8%). However, an important proportion of patients with MwA report other symptoms than visual as first occurring, although most of them report visual disturbances. This suggests that the origin of spreading depolarization might not be always the visual cortex, but also other brain regions (Bücking and Baumgartner, 1974; Viana et al., 2017). Furthermore, the pattern of symptom succession with subsequent visual or speech disturbance suggests spreading depolarization away but also toward the occipital region. Thus, relying on an expected symptom succession in differentiating migraine aura from incipient ischemic stroke might be misleading.

Occurrence of sensory and motor disturbance as first symptoms were more frequently described in ischemic stroke, with motor disturbance as first symptom having a high positive predictive value (88.9%). The parieto-occipital and rolandic sulci act as a natural barrier to the CSD in migraine aura, (Lauritzen et al., 1983; Hadjikhani et al., 2001) explaining the less frequently reported motor and sensory symptoms as first occurring. On the other hand, emboli in ischemic stroke may affect different regions than the occipital lobe typically involved in migraine aura (Lashley, 1941; Olesen et al., 1981; Cutrer et al., 1998). This makes it more likely that motor and sensory symptoms will be among the first to occur in ischemic stroke.

With a high positive predictive value (78.7%), the 5–60 min time-lapse between symptoms in migraine aura fits the currently established, although disputed (Bolay et al., 2019), theory of canonical CSD being the basis of migraine aura (Headache Classification Committee of the International Headache Society [IHS], 2018). Nevertheless, a substantial number of patients with migraine and consecutive symptoms reported at least two symptoms occurring simultaneously (19.4% in our cohort), not explained by the CSD theory. As previously discussed (Viana et al., 2017), there are insufficient data on the origin, propagation and areas affected by the CSD. Our findings support previous reports (Viana et al., 2017) suggesting heterogeneity of the mechanisms involved in the pathophysiology of migraine aura (Bolay et al., 2019).

As expected, in ischemic stroke with consecutive symptoms, at least two symptoms occurring simultaneously were more frequent (29%; PPV 46.6%). This finding is most probably due to ischemic core and penumbra. The latter also might explain why more stroke patients reported a time lapse between symptoms of < 1 min, albeit with a lower predictive value (PPV 57.1%). By contrast, a time lapse between symptoms of > 360 min appears to be highly predictive for ischemic stroke (100%). A longstanding well-compensated penumbral collateral circulation, hours later complicated by clot growth, clot rupture and distal migration are possible explanations (Rocha and Jovin, 2017).

Lastly, we identified visual symptoms consisting of complex visual hallucinations, certain patterns of spreading of sensory or motor symptoms, but also patterns of symptom succession that were reported only by patients with ischemic stroke or MwA (Supplementary Tables 1, 2, 5–8). One example is the so-called “cortical” pattern of spreading sensory symptoms (fingers→hand→mouth) and spreading within one confined anatomical region (e.g., from one finger to another) which has been reported by MwA patients only, in line with previous reports (Fisher, 1968; Bücking and Baumgartner, 1974).

Our study has strengths. First, the results are based on a structured live interview, minimizing the chance of misinterpretation of the questions. Second, although the questions were asked in a closed manner, there was a possibility for the participants of providing an open answer reducing the risk of suggestion. Third, the interviews with patients with stroke were performed soon after the index-event minimizing recall-bias. Forth, we interviewed a large patient cohort, thus reducing the risk of the results occurring by chance what makes the results more generalizable.

Our study has also limitations. Firstly, although the interviews were performed soon after the index-stroke, we cannot exclude a recall bias, especially in the migraine population, in which the delay was longer. Secondly, we did not systematically assess the cognitive function of our stroke participants and therefore we cannot exclude false reporting of symptoms because of covert cognitive deficits. During the interviews, there was no obvious cognitive deficit. Third, our findings are not generalizable to patients with severe stroke. Fourth, we did not assess the consistency of aura symptoms from aura to aura, nor the intraindividual aura variability. Fifth, the migraine and stroke groups were not matched with the stroke group being older with more males. Based on experimental data demonstrating a negative influence of age and male sex on the CSD propagation velocity, (Guedes et al., 2009; Guedes and Abadie-Guedes, 2019) assuming that ischemia-induced CSD explains at least partly the migraine-like symptoms in stroke, the migraine-like symptoms are likely underestimated in the stroke group. Sixth, the findings are not generalizable to patients with severe stroke, given the low median NIHSS (1, IQR 0–3).

In conclusion, even when analyzing the detailed content of patient’s history, symptoms occurring in ischemic stroke overlap to those of migraine aura. However, there are some elements of the history that seem to be characteristic for either conditions.

## Data availability statement

The raw data supporting the conclusions of this article will be made available by the authors, without undue reservation.

## Ethics statement

The studies involving human participants were reviewed and approved by Kantonale Ethikkommission Bern (KEK 2018-02258). The patients/participants provided their written informed consent to participate in this study.

## Author contributions

AS, CS, and SJ: study concept. AS, LK, and MB: data aquisition. JB, AS, CS, and SJ: data analysis. AS, CS, JB, and SJ: drafting of the manuscript. All authors contributed to critical revision of the article for intellectual content and approved the submitted version.
