# Supplementary material for: Symptoms and patterns of symptom propagation in incipient ischemic stroke and migraine aura
Source: Front Hum Neurosci. 2023 Jan 13;16:1077737. doi: 10.3389/fnhum.2022.1077737 (PMC9896624; doi:10.3389/fnhum.2022.1077737)
Supplement: Supplementary file 1 [file Data_Sheet_1.pdf]

Supplementary Table 1: Description of visual symptoms in patients with migraine who gave an additional answer to the predefined ones

Visual symptom	No of patients
Blind spot	27
Flickering	21
Bright spots	11
Visual Snow	9
Bright stripes	9
Reducing of the visual field	9
Everything was moving in a waved fashion	6
Sickle-shaped scintillation	3
Like neon light	3
Everything was seen through a piece of glass covered in milk	3
Bright stripes	3
Growing scintillating scotoma	3
Initially blindness, after few minutes scintillating scotoma	2
White dots	2
Blind spot with bright spikes around, distributed like a crown	2
Colored symmetric geometrical patterns; kaleidoscope	2
Like light reflecting on waving water	2
Small black points	2
Flickering point, which became a circle	2
Spider's web, bright stars	1
Different geometric patterns	1
A worm-like shape	1
Initially bright spots, which became blind spots after the brightness subsided	1
A bright worm-like shape, followed by a curtain, which left a blind area on one side of visual field of both eyes	1
Swooshy clouds	1
A red-green spot	1
A rainbow	1
Reducing of the visual field, colored waves	1
Everything looked golden	1
Colored triangles	1
Like bubbles in boiling water	1
Initially lightning, then something like star dust, then blind spot	1
Like diamantes	1
Horizontal flickering stripes	1
Like a spider's network with fine black parts	1
Colors, like a firework; on the right eye in the lower part of the visual field moving windmill of black and white color	1
Like a circle	1
Bright stripes and waves	1
Dark at first, then colored and bright	1
Ring-shaped silver light	1
Pattern with bright light	1
Silver colored light flashes	1
Bright triangle	1
A circle and a quadrant	1
Flashing and dark spots	1
Blurry vision, growing blind spot located at the periphery of the visual field, and transforms in a transparent sickle-shaped scintillation	1

Like a growing foldable mirror	1
Black circles, like a tunnel	1
White stripes	1
A flying sphere, also waves and scintillation; when reading, each letter must be read separately; the whole word at once cannot be read	1
Like a waterfall, blind spots, bright spots, no peripheral vision	1
Flickering rainbow	1
Growing kaleidoscope and a ring	1
Visual snow and black-white square patterns	1
On one half of visual field of both eyes dark vision; within the dark vision, bright stripes, like thunder	1
Small ring with bright and colored spikes around it	1
Colored points – green, blue and violet	1
Like a rotating rotor which shines in the sun	1
Blindness of one eye caused by scintillation, spontaneous neon light and stroboscope light	1
Ants that moved in all directions	1
Circles, triangles and clouds, which moved from right to left	1
Silver colored rotating waves	1
Foggy	1
Circle or half circle, oscillating	1
Initially a small black point, then a bright colored and zick-zack images	1
Yellow and orange colored spots, everything written looked smaller than usual	1
Like looking through a broken glass	1
Growing colored point	1
Blind colored red and green point	1
Like everything was in the fire	1
Everything was white	1
Bright and blind spots	1
Flickering, which became triangle followed by kaleidoscope	1
Like looking at the sun, on the periphery of visual field scintillating scotoma	1
Black points and stripes, but also golden stars	1
Visual snow on the periphery of visual field, in the center tunnel vision	1
On the peripheral site of both eyes cannot see anything	1
Spider’s web	1
Kaleidoscope	1
A bright flickering point	1
Diagonal stripe, blind spot on the left eye	1
Black and white triangles	1
Initially scintillating scotoma, then blurry	1
A white-black flickering	1
Bright stripes, additional lights like of a stroboscope	1
Circles, zig-zag lines, flickering, dark vision on both eyes on the right lower visual field	1
Growing bright stripes and pixelated bright scintillating points	1
Initially dark vision on both eyes on one half of the visual field, then growing scintillating scotoma	1
Tunnel vision and bright flashes	1
Visual snow, bright scintillation, bright flashes; bright grey over one half of the visual field	1
Multiple small circles	1
Moving black points, wave-like pattern	1
Moving spots	1

Flickering, dark vision on one side of the visual field	1
Like a curtain	1
Like bubbles in sparkling water	1
Like a circle with spikes, which comes close and goes far away	1
Circles	1
Bright form-like shapes	1
Double, triple, four till eight times the same objects were seen	1
Flickering and seeing double	1
Bright stripes, cannot see on one side of the visual field; within the blind visual field there is brightness	1
White scintillating	1
Bright stripes, flickering	1
Patterns of square figures	1
Colored circles and a blind spot	1
Grey scintillation	1

Supplementary Table 2: Description of visual disturbance in patients with ischemic stroke who gave additional answers.

bright stripes over entire visual field; the stripes moved in all directions
everything was inclined towards right, binocular
scintillating light, dark patches and a dark blue color seen with both eyes on the right side of visual field
a mixture of bright and dark patches, scintillating scotoma and intermittent visual field deficit in the upper right visual field, binocular
left monocular bright vision with bright brown patches
fixed dark points and scintillating scotoma on the left eye only
zig-zag lines, squared patterns over whole visual field
in the left hemianoptic visual field black-white dots, like a badly tuned analog old television
at first scintillating scotoma and zig-zag lines over the right visual field and afterwards hemianopsia towards right
right hemianopsia, within the anoptic visual field zig-zag lines, flickering and bright circles
initially colored Egyptian hieroglyphs, which moved from the peripheral visual field from both sides towards the center; after the hieroglyphs abated, the things around him became deformed, as if the patient would see a reflection of a broken mirror; additionally the things around him were moving in a wave-like pattern; after a while the patient saw a blue curtain or a carpet (not sure which one), which slowly moved from the periphery on both sides of the visual field towards the center; afterwards another ‘carpet’ emerged, this time in a pink color
‘snow storm’ and small transparent pixels in the right hemianoptic visual field
same object has been seen 4x times multiplied; each of the 4 images had a circle around it
everything was distorted, like looking through a broken glass
grey vision on right side of visual field
the left side of visual field was pink
colored fixed quadrants over entire visual field

Supplementary Table 3: Description of speech disturbance in patients with stroke who gave additional answers.

could not speak at all	21
spoke a ‘word salad’	6
babble	4
could not speak grammatically correctly	4
could not speak fluently and could not understand what others have said	3
could not speak fluently	2
could not calculate numbers	1
could not understand written text during reading	1
difficulty of modulating the voice	1
could not read or write	1
speaking required an inordinate amount of mental effort	1
always spoke the false words	1

Supplementary Table 4: Description of speech disturbance in patients with migraine aura who gave additional answers.

could not speak at all	19
could not speak grammatically correctly and could not understand what others have said	6
could not write correctly	5
could not read	5
could not speak grammatically correctly	5
said other words than intended	4
babble	3
could speak only few words	2
stuttered	2
could not read or write	2
word salad	1
the sentences were simplistic , spoke like a child	1
could only say one word	1
word salad and could not read	1
could not speak and write	1
could speak only slowly	1

Supplementary Table 5. Patterns of symptoms occurring in succession in ischemic stroke only

	**stroke, n/N (%)**
motor->speech	13/117 (11)
sensory->motor+speech	8/117 (7)
motor->sensory	6/117 (5)
speech->motor	5/117 (4)
sensory->motor->speech	3/117 (2)
speech+motor->sensory	2/117 (2)
sensory+speech->motor	2/117 (2)
visual->motor->sensory	1/117 (0.8)
visual->sensory+speech->motor	1/117 (0.8)
visual->motor->speech	1/117 (0.8)
sensory->speech->motor	1/117 (0.8)
speech->sensory+motor	1/117 (0.8)
speech->visual->sensory	1/117 (0.8)
speech->visual->motor	1/117 (0.8)
speech->motor->sensory	1/117 (0.8)
motor->speech->sensory	1/117 (0.8)
ataxia->sensory->motor	1/117 (0.8)
vertigo->speech	1/117 (0.8)
vertigo->sensory	1/117 (0.8)
vertigo->motor	1/117 (0.8)
visual+motor->speech	1/117 (0.8)
motor->not clear	1/117 (0.8)

Supplementary Table 6 Patterns of symptoms occurring in succession in migraine aura only

visual->sensory+speech	9/201 (4)
visual->speech->sensory	9/201 (4)
sensory->visual->speech	4/201 (2)
sensory->visual+speech	2/201 (0.9)
visual->vertigo	2/201 (0.9)
variable, without constant pattern	2/201 (0.9)
visual->sensory->motor->speech	1/201 (0.4)
visual->speech->sensory->motor	1/201 (0.4)
visual->variable symptoms	1/201 (0.4)
sensory->speech->visual	1/201 (0.4)
sensory->visual->motor->speech	1/201 (0.4)
speech->visual->sensory+motor	1/201 (0.4)
speech->visual+sensory	1/201 (0.4)
speech->motor+visual	1/201 (0.4)
motor->visual->speech	1/201 (0.4)
sensory+motor->speech->visual	1/201 (0.4)
visual+speech->sensory	1/201 (0.4)

Supplementary Table 7. Symptoms reported by patients with ischemic stroke only.

**Patterns of spreading of sensory disturbance**	n/N (%)
face->hand-> arm->leg	1/37 (3)
face->arm->torso->leg	1/37 (3)
face+tongue->arm	1/37 (3)
face+tongue->arm+leg	1/37 (3)
face->tongue->arm->leg	1/37 (3)
face->leg->arm	1/37 (3)
hand->face	1/37 (3)
forearm->upper arm->face+leg	1/37 (3)
arm->face->tongue->leg	1/37 (3)
hand->face->leg	1/37 (3)
upper arm->forearm->hand	1/37 (3)
arm->face->leg	1/37 (3)
upper arm->hand	1/37 (3)
leg->hand->face	1/37 (3)
foot->face	1/37 (3)
face+arm->leg	1/37 (3)
arm+leg->face	1/37 (3)
**Patterns of spreading of motor symptoms**	
leg->arm	7/37 (19)
arm->face	3/37 (8)
arm->leg	2/37 (5)
tongue->arm+leg	1/37 (3)
face->not clear	1/37 (3)
leg->arm->face->tongue	1/37 (3)
leg->hand+face	1/37 (3)
arm+leg->face	1/37 (3)

Supplementary Table 8. Symptoms reported by patients with migraine aura only.

**Patterns of spreading of sensory disturbance**	n/N (%)
face->arm	9/106 (8)
hand->arm	5/106 (5)
arm->face->leg	3/106 (3)
arm->face->tongue	2/106 (2)
hand->arm->face	2/106 (2)
fingers->arm>face	2/106 (2)
small finger->ring finger	2/106 (2)
small finger->ring finger->middle finger->first finger	2/106 (2)
face->arm->leg->tongue	1/106 (1)
around the eye->ear->cheek	1/106 (1)
head->torso->foot	1/106 (1)
tongue->small finger	1/106 (1)
fingers->hand->face->tongue	1/106 (1)
fingers->face->arm	1/106 (1)
fingers->whole hand	1/106 (1)
fingers->arm->leg->tongue->face	1/106 (1)
fingers->mouth	1/106 (1)
fingers->forearm->lips	1/106 (1)
fingers->forearm->upper arm->leg->face	1/106 (1)
small finger->ring finger->middle finger->first finger->thumb->tongue	1/106 (1)
small finger->ring finger->face	1/106 (1)
thumb->first finger->middle finger	1/106 (1)
hand->arm->face	1/106 (1)
hand->arm->leg	1/106 (1)
hand->forearm->upper arm->face->tongue	1/106 (1)
hand->forearm->upper arm->face	1/106 (1)
hand->forearm->upper arm	1/106 (1)
hand->shoulder->face	1/106 (1)
hand->lips->tongue	1/106 (1)
hand->face	1/106 (1)
hand->leg->face	1/106 (1)
hand->forearm->face	1/106 (1)
hand->forearm->upper arm->face	1/106 (1)
hand->elbow	1/106 (1)
hand->forearm	1/106 (1)
hand->face->leg->tongue	1/106 (1)
arm->face->tongue->leg	1/106 (1)
arm->torso->leg->face	1/106 (1)
arm->leg->torso->face	1/106 (1)
forearm->fingers->shoulder->face->neck->torso->leg	1/106 (1)
forearm->hand	1/106 (1)
**Patterns of spreading of motor symptoms**	
arm->face->leg	2/22 (9)
hand->face	1/22 (4)
fingers->whole hand	1/22 (4)
small and ring fingers->middle finger	1/22 (4)
face->arm	1/22 (4)
